# Supplementary material for: Extracellular vesicles from wild-type Epstein–Barr virus–transformed B-cells export host DNA and EBV EBER1
Source: bioRxiv. 2026 May 18:2026.03.30.715356. Preprint. [Version 2] doi: 10.64898/2026.03.30.715356 (PMC13317603; doi:10.64898/2026.03.30.715356)
Supplement: Supplement 1 [file media-1.pdf]

Supplemental Tables and Figures

Extracellular vesicles from wild-type Epstein–Barr virus–transformed B-cells export host DNA and EBV EBER1

Authors

Michelle L. Pleet[1,2], Rose Peterson[3], Stephanie Chidester[1], Emily H. Stack[2], Madeleine R. Druker[2], Jubin George[1], Charlotte V. Dagli[2], Abaigeal Donaldson[2], Joanna Palade[4], Elizabeth Hutchins[4], Chang-Sook Hong[1], Nyater Ngouth[2], Joan Ohayon[2], Maria Chiara Monaco[2], Jason Savage[1], Tapan K. Maity[5], Weiming Yang[5], Lisa M. Jenkins[5], Ru-Ching Hsia[6], Ionita C. Ghiran[7], Kendall van Keuren-Jensen[4], Kory Johnson[3], Jennifer C. Jones[1†*], Steven Jacobson[2†]

Supplemental Table 1: EBV proteins detected in SLCL-derived sEVs by DIA mass spectrometry.

Table listing EBV-encoded proteins and their associated descriptive names (Protein Description) identified in sEV preparations by DIA mass spectrometry across individual SLCL samples with corresponding abundance values. Positive (Namalwa) and negative (Ramos) control B-cell lines were included. Undetected proteins are denoted by " - ".

Protein	Protein Description	ND2	ND3	ND5	SMS2	SMS7	AMS2	AMS3	AMS4	Namalwa	Ramos
**MCP**	Major capsid protein	1.35E+04	4.15E+04	9.06E+04	3.04E+05	9.11E+04	2.37E+04	9.52E+04	1.48E+05	2.38E+04	-
**BLRF2**	Tegument protein BLRF2	-	-	5.49E+04	1.74E+05	1.32E+05	4.64E+04	5.12E+04	1.20E+05	-	-
**BBRF1**	Portal protein	-	-	2.27E+04	5.00E+04	2.08E+04	-	2.92E+04	1.01E+05	-	-
**BVRF2/BdRF1**	Capsid scaffolding protein	-	-	6.23E+04	1.81E+05	4.09E+04	1.52E+04	5.35E+04	9.63E+04	-	-
**BMRF1**	DNA polymerase processivity factor BMRF1	1.02E+04	-	4.88E+04	8.29E+04	4.78E+04	1.03E+04	6.43E+04	6.30E+04	-	-
**TRX2**	Triplex capsid protein 2	-	-	2.83E+04	9.88E+04	2.08E+04	4.28E+03	2.58E+04	4.67E+04	-	-
**RIR2**	Ribonucleoside-diphosphate reductase small subunit	1.23E+04	-	5.70E+04	3.31E+04	4.22E+04	1.34E+04	5.89E+04	4.48E+04	-	-
**BALF5**	DNA polymerase catalytic subunit	-	-	2.86E+04	3.70E+04	-	-	2.55E+04	3.95E+04	-	-
**gH**	Envelope glycoprotein H	-	-	3.57E+04	4.23E+04	2.95E+04	-	3.00E+04	3.83E+04	-	-
**RIR1**	Ribonucleoside-diphosphate reductase large subunit	1.77E+04	-	5.43E+04	4.36E+04	3.05E+04	1.81E+04	4.96E+04	3.75E+04	-	-
**BDLF3**	Glycoprotein BDLF3	-	-	1.82E+04	5.99E+04	2.02E+04	-	1.98E+04	3.11E+04	-	-
**gM**	Envelope glycoprotein M	-	-	5.04E+03	3.41E+04	7.04E+03	-	1.81E+04	2.92E+04	8.55E+02	-
**BGLF4**	Serine/threonine-protein kinase BGLF4	-	-	1.89E+04	2.54E+04	2.52E+04	-	2.06E+04	2.28E+04	-	-
**dUTPase**	Deoxyuridine 5'-triphosphate nucleotidohydrolase	5.06E+03	-	1.84E+04	2.01E+04	2.35E+04	-	2.95E+04	2.21E+04	-	-
**TRX1**	Triplex capsid protein 1	-	-	7.97E+03	3.77E+04	9.30E+03	-	1.09E+04	2.12E+04	-	-
**SCP**	Small capsomere-interacting protein	-	-	-	4.59E+04	-	-	2.42E+04	1.60E+04	-	-
**BMRF2**	Protein BMRF2	-	-	1.27E+04	3.25E+04	-	-	-	1.47E+04	-	-
**UNG**	Uracil-DNA glycosylase	-	-	4.49E+04	-	-	-	3.36E+04	1.38E+04	-	-
**LMP2**	Latent membrane protein 2	1.68E+04	1.52E+04	1.40E+04	1.98E+04	9.04E+03	1.80E+04	1.60E+04	1.38E+04	-	-
**BNRF1**	Major tegument protein	-	-	6.51E+03	2.60E+04	8.10E+03	-	1.21E+04	1.21E+04	-	-
**EBNA-LP**	Epstein-Barr nuclear antigen leader protein	-	-	1.29E+04	4.18E+03	1.43E+04	2.27E+04	3.93E+04	1.21E+04	-	-
**LF2**	Protein LF2	-	-	-	1.28E+04	-	-	9.19E+03	1.10E+04	-	-
**gB**	Envelope glycoprotein B	-	-	5.18E+03	2.55E+04	4.63E+03	-	9.28E+03	1.08E+04	-	-
**EBNA1**	Epstein-Barr nuclear antigen 1	3.13E+03	6.17E+03	2.40E+03	5.53E+03	-	2.66E+03	4.94E+03	8.97E+03	-	-
**BDLF2**	Protein BDLF2	-	-	4.21E+03	1.34E+04	5.60E+03	-	8.72E+03	7.41E+03	-	-
**BNLF2b**	Uncharacterized protein BNLF2b	-	-	3.30E+03	7.66E+03	3.76E+03	-	3.67E+03	6.89E+03	-	-
**BMLF1**	mRNA export factor ICP27 homolog	5.99E+02	-	1.17E+04	2.93E+03	5.69E+03	-	1.03E+04	6.55E+03	-	-
**BGLF5**	Shutoff alkaline exonuclease	1.56E+03	-	1.44E+04	6.89E+03	8.11E+03	-	1.03E+04	5.55E+03	-	-
**BARF1**	Secreted protein BARF1	-	-	9.40E+03	1.16E+04	5.41E+03	-	1.13E+04	5.35E+03	-	-
**gH**	Envelope glycoprotein H	-	-	-	-	-	-	-	3.79E+03	-	-
**BRRF1**	Transcriptional activator BRRF1	-	-	1.38E+04	3.29E+03	5.11E+03	-	4.52E+03	3.58E+03	-	-
**TK**	Thymidine kinase	-	-	4.13E+03	-	-	-	4.20E+03	2.78E+03	-	-
**BHRF1**	Apoptosis regulator BHRF1	-	-	1.72E+03	1.74E+03	9.99E+02	-	1.49E+03	2.71E+03	-	-
**gL**	Envelope glycoprotein L	-	-	1.22E+03	3.42E+03	-	-	5.52E+03	2.51E+03	-	-
**BRRF2**	Tegument protein BRRF2	-	-	-	1.75E+03	-	-	1.88E+03	1.54E+03	-	-
**BRLF1**	Replication and transcription activator	-	-	1.43E+03	-	-	-	1.38E+03	1.32E+03	-	-
**BZLF2**	Glycoprotein 42	-	-	2.22E+03	1.80E+03	1.88E+03	-	2.04E+03	9.57E+02	-	-
**BALF1**	Apoptosis regulator BALF1	-	-	-	4.23E+03	-	-	-	-	-	-
**BFLF1**	Packaging protein UL32 homolog	-	-	5.19E+03	1.08E+03	-	-	3.95E+03	-	-	-
**CVC2**	Capsid vertex component 2	-	-	-	1.94E+04	-	-	-	-	-	-

Supplemental Table 2: EBV RNA counts detected in SLCL-derived sEVs by total stranded RNA sequencing.

Table listing EBV-encoded transcripts detected from SLCL sEV RNASeq, with counts for each transcript across individual SLCL samples and comparator lines where indicated. EBER1 was the most abundant EBV transcript across sEV preparations. EBV positive control cell line Namalwa was included. Undetected RNAs are denoted by “ - ”.

Product	Namalwa	ND2	ND3	ND5	SMS2	SMS7	AMS2	AMS3	AMS4
EBER-1	234	1489	3197	1960	1260	3788	4049	4777	276
EBNA-3B	218	33	167	1171	447	836	398	1139	584
LMP-1	-	6	46	1947	423	441	451	845	520
RPMS1	-	184	62	896	513	510	182	1146	457
EBNA-3C	218	23	144	854	266	650	377	910	436
EBNA-2	294	76	138	716	515	729	258	810	267
EBNA-3A	218	24	129	753	364	454	237	885	486
EBNA-1.1	218	21	130	504	90	475	179	605	263
A73	-	134	34	530	394	196	99	642	230
ebv-miR-BHRF1-3	-	62	45	653	456	251	27	486	168
EBNA-LP	211	53	80	434	124	413	185	599	111
Rta	1	17	6	489	177	373	82	403	322
EBER-2	17	55	307	275	127	221	117	284	50
LMP-2B	15	8	82	189	120	108	71	162	347
EBNA-1	9	-	8	273	97	173	24	232	93
Zta	-	-	-	265	69	91	77	204	106
ebv-miR-BHRF1-2	-	12	-	302	167	54	24	172	47
ebv-miR-BHRF1-2*	-	12	-	166	143	60	24	121	54
LMP-2A	-	2	-	102	27	53	-	150	110
ebv-miR-BART17-5p	-	-	-	-	51	16	-	90	22
ebv-miR-BART15	-	-	-	-	8	10	23	98	35
ebv-miR-BART6-5p	-	-	-	-	35	27	-	91	10
ebv-miR-BART6-3p	-	-	1	1	5	30	-	84	17
ebv-miR-BART17-3p	-	-	-	-	35	16	-	71	14
ebv-miR-BART16	-	9	-	-	2	7	-	78	19
ebv-miR-BART14*	-	-	-	38	5	14	-	16	23
ebv-miR-BART5*	-	-	-	-	5	-	-	55	15
RPMS1-25	-	-	-	5	11	1	-	15	31
ebv-miR-BHRF1-1	-	-	-	20	1	7	-	23	9
ebv-miR-BART5	-	-	-	-	2	-	-	39	10
ebv-miR-BART13*	-	-	4	3	-	23	-	11	8
ebv-miR-BART9	-	-	-	-	-	-	-	-	43
ebv-miR-BART9*	-	-	-	1	-	-	-	-	41
ebv-miR-BART8	-	-	-	1	-	1	-	14	22
ebv-miR-BART13	-	-	-	-	-	20	-	11	4
ebv-miR-BART19-5p	-	-	-	-	-	-	-	12	19
ebv-miR-BART4*	-	-	2	1	10	-	10	-	8
ebv-miR-BART3	-	-	3	2	-	-	10	8	5
ebv-miR-BART10*	-	-	-	1	1	5	-	-	18
ebv-miR-BART8*	-	-	-	-	-	-	2	-	23
ebv-miR-BART18-3p	-	-	-	18	-	-	1	2	1
ebv-miR-BART12	-	-	-	18	-	-	-	2	1
ebv-miR-BART11-5p	-	-	-	-	-	-	-	-	20
ebv-miR-BART10	-	-	-	-	-	-	-	-	19
ebv-miR-BART14	-	-	-	-	-	-	-	2	17
ebv-miR-BART11-3p	-	-	-	-	-	-	-	-	18
ebv-miR-BART21-5p	-	-	-	-	1	3	2	2	9
ebv-miR-BART4	-	-	-	-	10	-	-	1	6
ebv-miR-BART19-3p	-	-	-	-	-	-	-	-	15
ebv-miR-BART2-5p	-	-	-	-	1	1	-	-	11
ebv-miR-BART20-3p	-	-	-	4	2	-	-	-	5
ebv-miR-BART2-3p	-	-	-	-	-	-	1	-	10
ebv-miR-BART1-3p	-	-	-	1	2	-	-	1	5
ebv-miR-BART1-5p	-	-	-	1	-	-	-	2	3
ebv-miR-BART20-5p	-	-	-	-	-	-	-	1	5
ebv-miR-BART7*	-	-	-	-	-	-	-	-	6
ebv-miR-BART3*	-	-	3	-	-	-	-	-	-
ebv-miR-BART21-3p	-	-	-	-	-	-	-	-	2
ebv-miR-BART7	-	-	-	-	-	-	-	-	1

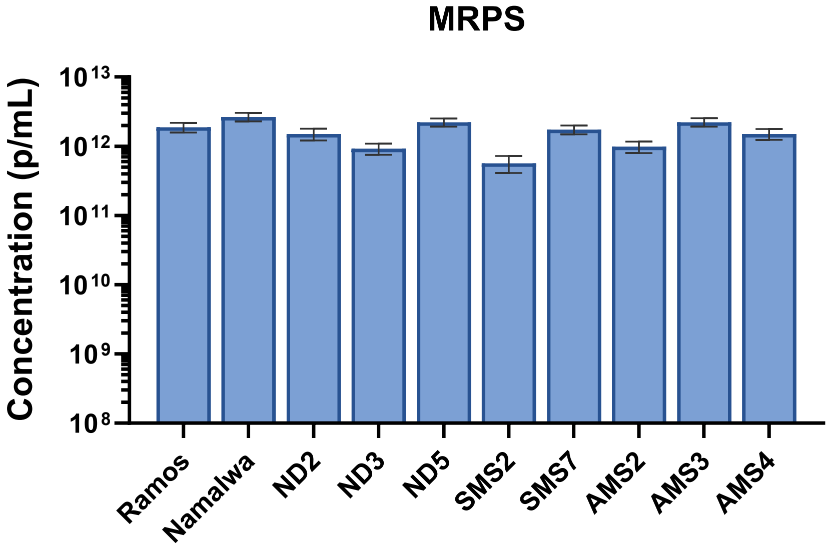

Supplemental Figure 1: MRPS quantification of sEV preparations from SLCLs

Microfluidic resistive pulse sensing (MRPS) quantification of sEV concentration in particles/mL (p/mL) generated from normal donor (ND), stable MS (SMS), and active MS (AMS) SLCLs. Bars represent mean particle concentration.

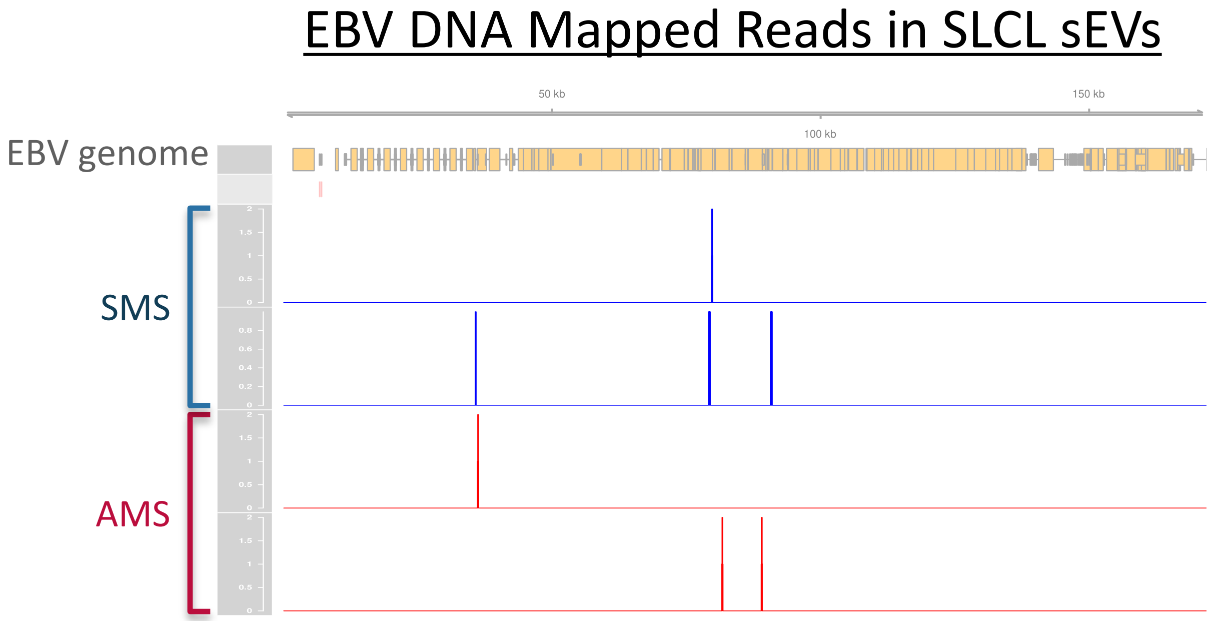

Supplemental Figure 2: EBV genomic DNA is minimally represented in SLCL-derived sEVs. Integrated Genome Viewer (IGV) browser view of DNASeq reads mapped to the EBV genome from SLCL-derived sEV preparations. Across few SMS and AMS samples, only sparse and discontinuous EBV-aligned reads were detected, with no consistent enrichment across specific viral genomic regions.

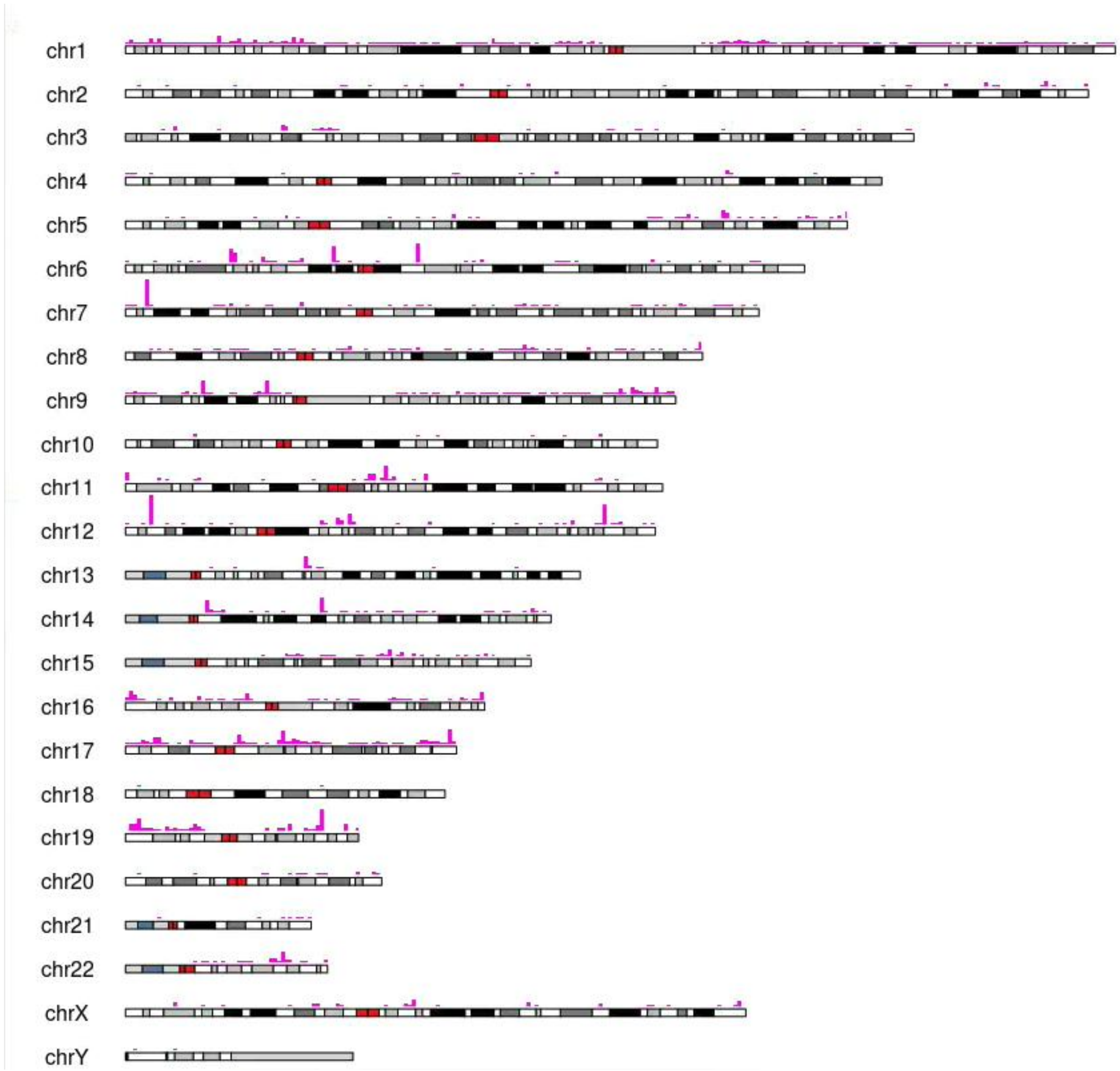

Supplemental Figure 3: Representative chromosomal mapping of host RNA reads in SLCL-derived sEVs.

Representative karyotype density plot showing genome-wide distribution of host-derived SLCL sEV RNASeq reads (magenta vertical bars) across human chromosomes. Reads are distributed broadly throughout the genome without a dominant positional bias. Red blocks show centromeric regions of each chromosome. Black and light-gray blocks represent the chromosome ideogram/cytoband structure.

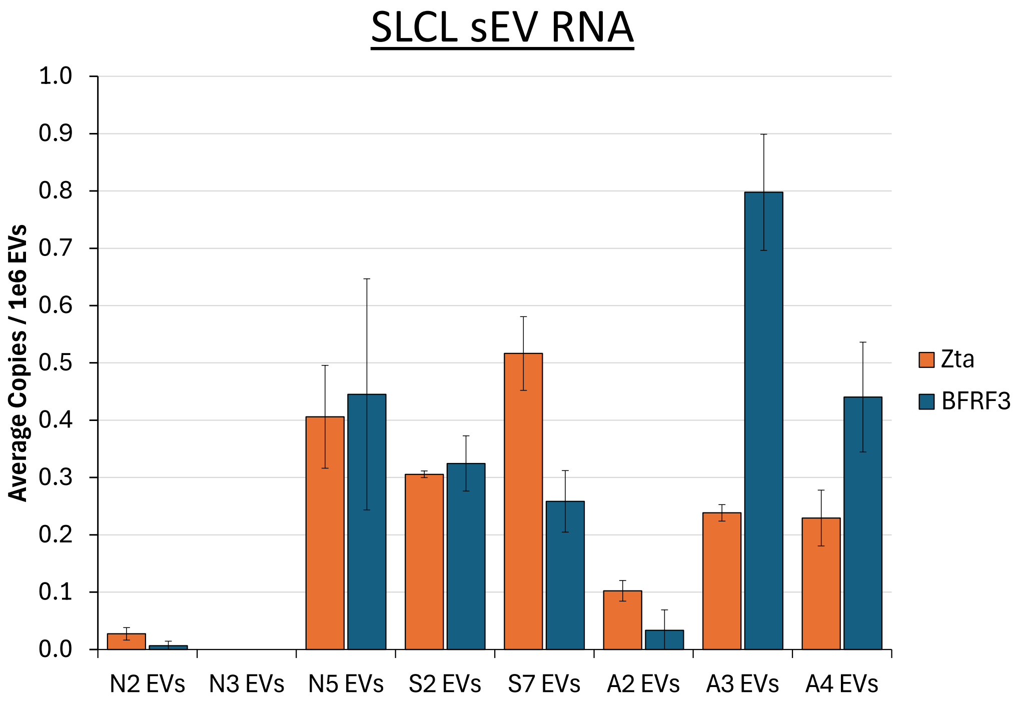

Supplemental Figure 4: Detection of low-abundance lytic EBV transcripts in SLCL-derived sEV RNA.

Droplet digital PCR quantification of the lytic EBV transcripts BZLF1 (Zta; orange) and BFRF3 (blue) from sEV RNA from individual SLCL lines, reported as average copies per 10^6 EVs.

A)

DIA Proteomics sEV Analysis Pipeline

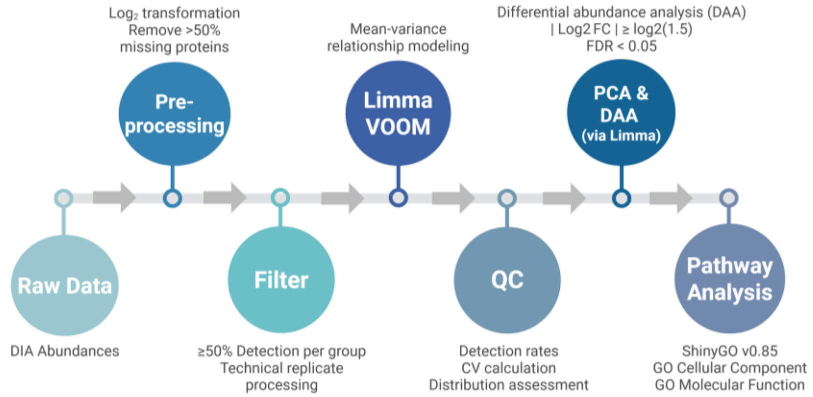

B)

WGS DNASeq Human sEV Analysis Pipeline

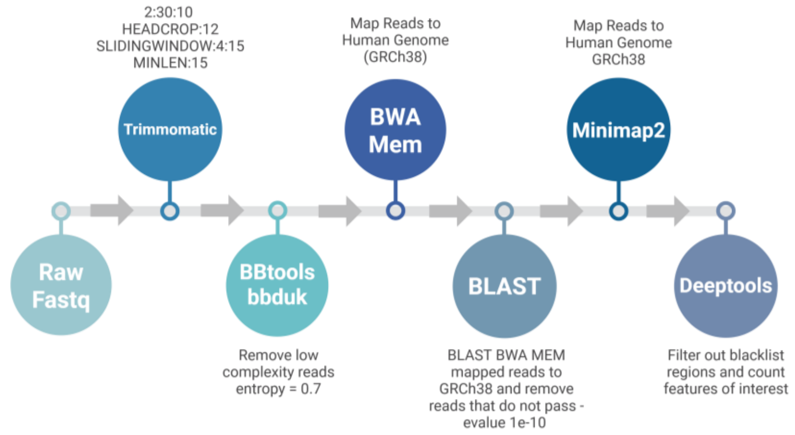

C)

Total Stranded RNASeq Human sEV Analysis Pipeline

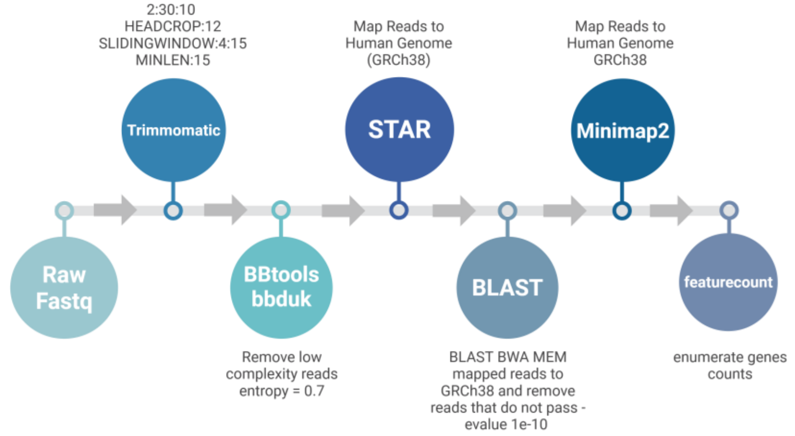

Supplemental Figure 5: Bioinformatic pipelines used for multiomic analysis of SLCL-derived sEV cargo.

A) Schematic of the DIA proteomics analysis workflow, including preprocessing, filtering, normalization with limma/voom, quality control, differential expression analysis, and pathway enrichment analysis. **B)** Schematic of the whole-genome sequencing human DNA analysis pipeline, including adapter and quality trimming, low-complexity filtering, alignment to GRCh38, BLAST-based validation, remapping, and downstream quantification/visualization. **C)** Schematic of the total stranded RNASeq human analysis pipeline, including read trimming, low-complexity filtering, alignment to GRCh38 with STAR, BLAST-based validation, remapping, and downstream quantification/visualization. Pipeline figures were created using BioRender.
